# Increased number of cryptosporidiosis cases with travel history to Croatia might be related to swimming pools, Germany, 2023

**DOI:** 10.2807/1560-7917.ES.2024.29.1.2300699

**Published:** 2024-01-04

**Authors:** Anja Schoeps, Klara Röbl, Nicole Walter, Andrea Neute, Bernadette Walter, Inga Freudenau, Annette Jurke, Christiane Klier, Petra Heinmüller, Syamend Saeed, Jasmin Metz, Hendrik Wilking, Philipp Zanger

**Affiliations:** 1 Heidelberg Institute of Global Health (HIGH), Heidelberg University, Heidelberg, Germany; 2 Federal State Agency for Consumer and Health Protection Rhineland-Palatinate, Koblenz, Germany; 3 Postgraduate Training for Applied Epidemiology (PAE), Department of Infectious Disease Epidemiology, Robert Koch Institute, Germany; 4 District Public Health Authority Rhein-Pfalz-Kreis, Ludwigshafen, Germany; 5 Landesamt für Arbeitsschutz, Verbraucherschutz und Gesundheit (LAVG), Potsdam, Germany; 6 Department for Health Protection, Infection Control and Epidemiology, State Health Office, Ministry of Social Affairs, Health and Integration Baden-Württemberg, Stuttgart, Germany; 7 Landeszentrum Gesundheit Nordrhein-Westfalen, Fachgruppe Infektionsepidemiologie, Bochum, Germany; 8 Niedersächsisches Landesgesundheitsamt, Abt. 2: Infektionsepidemiologie, Hannover, Germany; 9 Hessisches Landesamt für Gesundheit und Pflege (HLfGP), Abteilung 2 Gesundheits- und Infektionsschutz, Dillenburg, Germany; 10 Bayerisches Landesamt für Gesundheit und Lebensmittelsicherheit, München, Germany; 11 Department of Infectious Disease Epidemiology, Robert Koch Institute, Germany; * These authors contributed equally to this work and share first authorship.

**Keywords:** cryptosporidiosis, *Cryptosporidium* spp, swimming pools, travel, surveillance, outbreak

## Abstract

In August and September 2023, an unusually high number of cryptosporidiosis cases identified by routine German surveillance had travelled to Croatia (n = 23). Nine cases had stayed in the same camping resort and seven further cases had stayed at other camping sites within 15 km. Based on our standardised questionnaires, the most likely source of infection was swimming pools (93%). Further environmental investigations on site might reveal potential common sources of contamination that could be targeted by control measures.

On 25 September 2023, the federal state public health authority of Rhineland-Palatinate was informed by one local health authority about one laboratory-confirmed case of cryptosporidiosis (index case) and three epidemiologically linked cases, who had all spent a vacation in a camping resort in Istria, Croatia, during their incubation period (1–12 days). There were further anecdotal reports of cases with similar symptoms among other members of this travel group.

Cryptosporidiosis is characterised by watery diarrhoea, nausea, vomiting, abdominal pain and fever. Past outbreaks in Europe and the United States have been associated with contaminated food or drink and with recreational water [1-4]. The aims of this investigation were (i) to a assess whether there was a significant increase in cryptosporidiosis cases with potential exposure in Croatia and (ii) to identify the most likely cause of infection using information from case interviews.

## Case numbers by country of exposure

An investigation in the German national notification database revealed 23 cryptosporidiosis cases with potential exposure in Croatia notified between calendar week 31 and 39 (31 July to 1 October 2023). In accordance with the German infectious diseases surveillance system, a cryptosporidiosis case was defined as either a patient with a laboratory diagnosis of *Cryptosporidium* spp. (antigen testing or PCR or stool microscopy) and clinical symptoms of cryptosporidiosis (abdominal pain or diarrhoea or death) *or* a patient with those symptoms and an epidemiological link to a laboratory-confirmed case [5]. Country of exposure was assessed by local health authorities during routine case investigation, which is initiated for every reported cryptosporidiosis case.

From calendar weeks 31 to 39, 2023, we found cryptosporidiosis cases in Germany with exposure in Croatia to be particularly numerous: 23 cases compared with between zero and four cases in the previous 5 years (Table 1). During the same period, the overall number of cryptosporidiosis cases in Germany also showed a significant increase: from 600–750 cases before the COVID-19 pandemic in 2018 and 2019 and 400–550 cases between 2020 and 2022 to 1,001 cases in 2023 (p < 0.001). This overall increase may have been caused by a more frequent use of multiplex PCR for *Cryptosporidium* diagnostics: ca 15% of cryptosporidiosis cases were diagnosed by PCR in 2018 and 2019, compared with ca 50% in 2023.

**Table 1:** Cases notified with cryptosporidiosis in Germany, calendar week 31–39 in 2018–2023: overall, with exposure in southern Europe^a^, and with exposure in Croatia (n = 3,855)

	All cases	Southern Europe^a^ (excluding Croatia)				Croatia			
	n	n	%	Odds	OR^b^ (95% CI)	n	%	Odds	OR^b^ (95% CI)
2018	758	46	6.1	0.07^c^	Reference	1	0.1	0.00^c^	Reference
2019	607	41	6.8			3	0.5		
2020	410	12	2.9	0.03^c^	0.42 (0.26–0.67)^c^	3	0.7	0.01^c^	2.47 (0.63–11.56)^c^
2021	524	14	2.7			4	0.8		
2022	555	26	4.7	0.05	0.72 (0.44–1.14)	0	0.0	0.00	0.00 (0.00–2.32)
2023	1,001	87	8.7	0.10	1.43 (1.04–1.97)	23	2.3	0.03	8.22 (2.79–32.79)

CI: confidence interval; OR: odds ratio.

^a^ France, Greece, Italy, Portugal and Spain.

^b^ Ratio of odds of exposure abroad among cases notified with cryptosporidiosis in the combined years 2020 and 2021, in 2022 and in 2023, respectively vs baseline of the combined years 2018 and 2019.

^c^ 2018/19 and 2020/21 combined, respectively.

To verify that the data for Croatia were not explained by the general increase in cryptosporidiosis cases in 2023, we calculated odds ratios (OR) of exposures with 95% confidence intervals (CI). To obtain a reliable reference, the years before the COVID-19 pandemic 2018 and 2019 were combined. Likewise, the pandemic years 2020 and 2021 were combined. Among notified cryptosporidiosis cases in Germany, the odds of exposure in Croatia were significantly higher in 2023 than in 2018 and 2019 (OR = 8.22; 95% CI: 2.79–32.79). The odds of exposure in southern Europe (France, Greece, Italy, Portugal and Spain) were also increased, but by a much lower magnitude (OR = 1.43; 95% CI: 1.04–1.97), indicating an effect specific for exposure in Croatia rather than a general trend.

## Exposure assessment

The 23 cases with potential exposure in Croatia between weeks 31 and 39 had symptom onset between weeks 28 and 37 and were reported by seven German federal states: Baden-Württemberg (five cases), Brandenburg (five cases), Rhineland-Palatinate (four cases), North Rhine-Westphalia (three cases), Bavaria (two cases), Hesse (two cases) and Lower Saxony (two cases). The cases’ median age was 8 years, with a range from 1 to 48 years, and sex was distributed evenly between male and female (Table 2). 

**Table 2:** Cryptosporidiosis cases with exposure in Croatia, notified in Germany, 2023 (n = 23)

ID	Reporting week	Location of exposure	Distance to Camping 1^a^ (km)	Pool used	Type of confirmation^b^	Questionnaire returned	Age group (years)	Sex
01^c^	38	Camping 1	0	Yes	Laboratory	Yes	5–9	M
02	39	Camping 1	0	Yes	Epidemiology	Yes	5–9	F
03	39	Camping 1	0	Yes	Epidemiology	Yes	40–49	M
04	39	Camping 1	0	Yes	Epidemiology	Yes	30–39	F
05	37	Camping 1	0	?	Laboratory	No	20–29	F
06	37	Camping 1	0	?	Epidemiology	No	0–4	F
07	37	Camping 1	0	?	Epidemiology	No	0–4	M
08	37	Camping 1	0	?	Epidemiology	No	30–39	M
09	33	Camping 1	0	Yes	Laboratory	Yes	10–19	F
10	35	Camping 2	1	Yes	Laboratory	Yes	10–19	M
11	39	Camping 3	2	Yes	Laboratory	Yes	0–4	M
12	37	Camping 4	3	Yes	Laboratory	Yes	0–4	F
13	38	Camping 5^d^	13	Yes	Laboratory	Yes	0–4	F
14	33	Camping 5	13	?	Laboratory	No	5–9	M
15	33	Camping 5	13	?	Laboratory	No	5–9	F
16	36	Camping 6	14	Yes	Laboratory	Yes	0–4	M
17	35	Camping 7	20	Yes	Laboratory	Yes	0–4	F
18	38	Camping 8	32	Yes	Laboratory	Yes	10–19	F
19	31	Camping 9	34	No	Laboratory	Yes	40–49	M
20	36	Camping 10	> 100	Yes	Laboratory	Yes	10–19	M
21	36	Camping 10	> 100	Yes	Laboratory	Yes	30–39	F
22	39	NA^e^	NA^e^	NA^e^	Laboratory	No	0–4	M
23	32	NA^e^	NA^e^	NA^e^	Laboratory	No	5–9	M

F: female; ID: identification number; M: male; NA: not available; ?: unknown.

^a^ Euclidean distance.

^b^ Case definition is confirmed if (i) clinical criteria (one out of abdominal pain or diarrhoea or death due to cryptosporidiosis) and laboratory-testing (antigen-testing, polymerase-chain-reaction (PCR), stool microscopy) are met or if (ii) clinical criteria in a case with link to another laboratory-confirmed case are present [5].

^c^ Index case.

^d^ Bungalow next to Camping 5.

^e^ This information is not available because cases could not be contacted by the health authorities.

Information regarding places of potential exposure in Croatia was available for 21 of 23 cases: They had stayed at 10 different camping resorts, of which nine are located in Istria. Twelve cases stayed either at the camping resort of the index case or in close vicinity of 3 km or less (Figure). We notified the Croatian public health authorities via the European Early Warning and Response System about the initial findings and suspected places of exposure on 6 October 2023.

**Figure:**
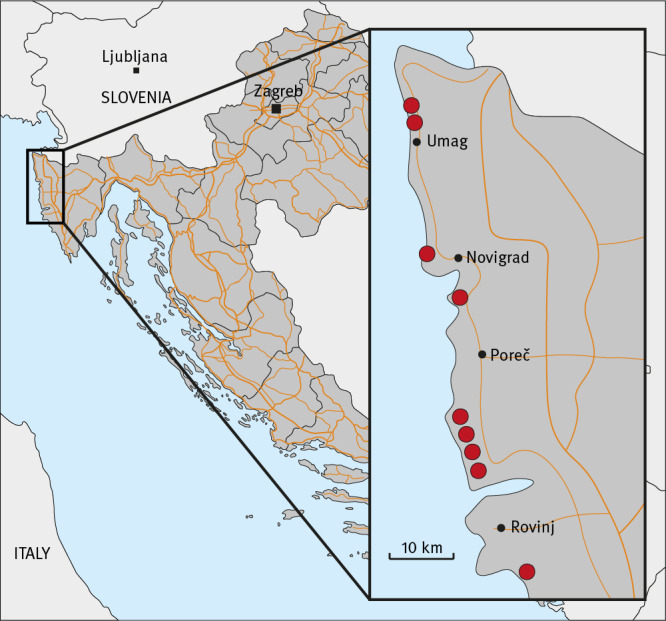
Locations of Istrian camping sites with at least one cryptosporidiosis case later identified in Germany, 2023 (n = 19 cases)

One camping resort outside Istria is not shown.

We developed a structured questionnaire to comprehensively assess potential sources of infection, which was administered by state or local health authorities. All 23 cases (or the legal representative if the case was a child) were invited to participate, and 15 completed questionnaires were returned.

All cases had experienced at least two cryptosporidiosis-related symptoms, and 12 of 14 cases had cancelled planned activities due to illness (Table 3). Fourteen of 15 respondents said they used pools at the camping resorts where they had stayed. Only two cases reported that they also swam in other pools or freshwater during their vacation. The only case without exposure to a pool reported contact to a symptomatic person within 14 days before disease onset. Twelve of 15 participants reported swimming in the sea. Five of 13 cases reported that they used tap water to brush their teeth and none of the cases had drunk tap water without boiling it. Among potentially contaminated food items, consumption of raw unpeeled fruit (eight of 13 cases), raw unpeeled vegetables (seven of 13 cases) and ice cream sold openly (six of 14 cases) was reported most frequently, whereas consumption of raw meat or fish, raw milk or ice cubes was stated in fewer instances.

**Table 3:** Symptoms and associated factors for notified cryptosporidiosis cases with questionnaire returned, Germany, 2023 (n = 15)

	Yes	No	Unknown
Symptoms			
Diarrhoea	13	2	0
Stomach ache	15	0	0
Vomiting	6	8	1
Fever ≥ 38 °C	7	7	1
General malaise	13	2	0
Condition affected daily activities	13	1	1
Daily activities cancelled	12	2	1
Potential risk factors			
Swimming in camping site pool	14	1	0
Swimming in other freshwater	2	12	1
Swimming in sea	12	3	0
Drinking water from tap/fountain	0	14	1
Using tap/fountain water for brushing teeth	5	8	2
Drinks with ice cubes	4	9	2
Raw unpeeled fruit	8	5	2
Raw vegetables	7	6	2
Raw meat/fish	4	9	2
Unpasteurised milk	2	11	2
Ice cream sold openly	6	8	1
Animal contact	2	13	0
Previous contact with symptomatic person	5	7	3

## Discussion

Similar to reports from the United Kingdom and Ireland [6,7], we observed a marked increase in cryptosporidiosis cases in Germany during Summer 2023 when compared with the same period in previous years. Simultaneously, we found that the proportion of cases in Germany with a travel history to Croatia had increased significantly in 2023. As the majority of cases with reported potential exposure in Croatia had stayed on camping sites located in close vicinity of less than 15 km in Istria, these data suggest transmission of *Cryptosporidium* spp. in Istria during Summer 2023. In communication with public health experts in some of Germany’s neighbouring countries, we became aware of three additional cryptosporidiosis cases reported in Luxembourg, who also had a travel history to Croatia (personal communication: Corinna Ernst, Health Directorate, Strassen, Luxembourg, 13 December 2023). One of the Luxembourg cases had stayed at Camping 1 as well and one case in close vicinity, while the third case did not provide information about the camping site.

All but one respondent reported exposure to swimming pools. This points towards potential acquisition of cryptosporidiosis through ingestion of contaminated water. *Cryptosporidium* spp. oocysts are resistant to chlorine [8] and an association between swimming in recreational waters and cryptosporidiosis has been described in multiple studies [4,9-11]. A systematic review of worldwide outbreaks found recreational waters and swimming pools to be the source in 75.2% of studied waterborne parasitic outbreaks and in 92% of studied waterborne cryptosporidiosis outbreaks [11]. Since a large number of participants also reported swimming in the sea, exposure to *Cryptosporidium* spp. in maritime water might be an alternative source of infection among the investigated cases, although the literature has fewer reports about *Cryptosporidium* spp. infection through exposure to sea water than recreational water. There are generally more *Cryptosporidium* spp. in swimming pools than in marine water in Europe [12].

Heavy rainfalls in early August 2023 in the area where cases had stayed [13] could have caused water contamination. However, more than one quarter of cases had already experienced symptoms before the rainfall (data not shown). Biweekly analyses and monitoring of the sea water quality on Croatian beaches reported excellent water quality in Istria throughout Summer 2023 [14]. Although *Cryptosporidium* spp. was not measured directly, intestinal enterococci and *Escherichia coli* may serve as surrogate markers for potential faecal contamination. Other possible sources of infection, such as animal contact, consumption of contaminated food or person-to-person transmission are not supported by our findings.

We found that most cases had to cancel daily activities due to cryptosporidiosis, which shows the pathogenic potential of *Cryptosporidium* spp. It also supports probable under-reporting by mandatory notification, because the outbreak most probably also included a larger number of mild cases, which were not captured in routine surveillance because they did not come into contact with the healthcare system. Thus, the public health risk associated with *Cryptosporidium* spp., is most probably underrated, as reported elsewhere [1].

Our study had the following limitations: Firstly, we were unable to determine which *Cryptosporidium* species caused illness in travellers returning from Croatia. Laboratory diagnosis of cryptosporidiosis in Germany is normally based on antigen testing or PCR, where most conventional PCR kits do not differentiate between species. Moreover, several attempts to obtain additional stool samples for species diagnosis failed. This is unfortunate since species differentiation gives important clues to whether a zoonotic source must be considered. Secondly, we are not aware of any increase in cryptosporidiosis cases reported from the Croatian authorities for Summer 2023. However, the swimming pools at the camping sites were probably most popular among tourists, so a contamination of swimming pool water might not have caused a high number of cases in the Croatian population. In addition, based on the large differences in reported cryptosporidiosis cases across Europe [15], we hypothesise that the degree of underdiagnosis and under-reporting of cryptosporidiosis varies between countries [1]. Thus, the absence of a signal in Croatia might be explained by these mechanisms, rather than by a fault in our data or a chance result. Finally, we acknowledge that both the small sample size and the lack of a control group somewhat weaken our hypothesis of cryptosporidiosis infection by ingestion of recreational water on camping sites.

## Conclusion

We observed an increased risk for *Cryptosporidium* spp. infection among German travellers returning from the west coast of Istria in Summer 2023, probably acquired in swimming pools. A local environmental investigation could test this hypothesis and identify a possible common source of *Cryptosporidium* spp. contamination of recreational water to be considered ahead of the summer 2024. International cooperation among countries in the European Union regarding cryptosporidiosis surveillance should be strengthened and the awareness about the risk of acquiring cryptosporidiosis from recreational waters should be enhanced among the European population.
